# LRF-WiVi: A WiFi and Visual Indoor Localization Method Based on Low-Rank Fusion

**DOI:** 10.3390/s22228821

**Published:** 2022-11-15

**Authors:** Wen Liu, Changyan Qin, Zhongliang Deng, Haoyue Jiang

**Affiliations:** School of Electronic Engineering, Beijing University of Posts and Telecommunications, Beijing 100876, China

**Keywords:** fingerprint localization, WiFi channel state information, visual images, low rank fusion, end-to-end

## Abstract

In this paper, a WiFi and visual fingerprint localization model based on low-rank fusion (LRF-WiVi) is proposed, which makes full use of the complementarity of heterogeneous signals by modeling both the signal-specific actions and interaction of location information in the two signals end-to-end. Firstly, two feature extraction subnetworks are designed to extract the feature vectors containing location information of WiFi channel state information (CSI) and multi-directional visual images respectively. Then, the low-rank fusion module efficiently aggregates the specific actions and interactions of the two feature vectors while maintaining low computational complexity. The fusion features obtained are used for position estimation; In addition, for the CSI feature extraction subnetwork, we designed a novel construction method of CSI time-frequency characteristic map and a double-branch CNN structure to extract features. LRF-WiVi jointly learns the parameters of each module under the guidance of the same loss function, making the whole model more consistent with the goal of fusion localization. Extensive experiments are conducted in a complex laboratory and an open hall to verify the superior performance of LRF-WiVi in utilizing WiFi and visual signal complementarity. The results show that our method achieves more advanced positioning performance than other methods in both scenarios.

## 1. Introduction

Due to the complexity and variability of the indoor environment, high-quality indoor location-based services are challenging to be implemented using a single signal source. Multi-source fusion has become an important research trend in the field of indoor localization [1]. As two low-cost and easy-to-obtain signals, WiFi and vision contain complementary location information. The combination of the two signals is expected to improve positioning accuracy and stability in complex indoor environments [2].

**Figure 1:**
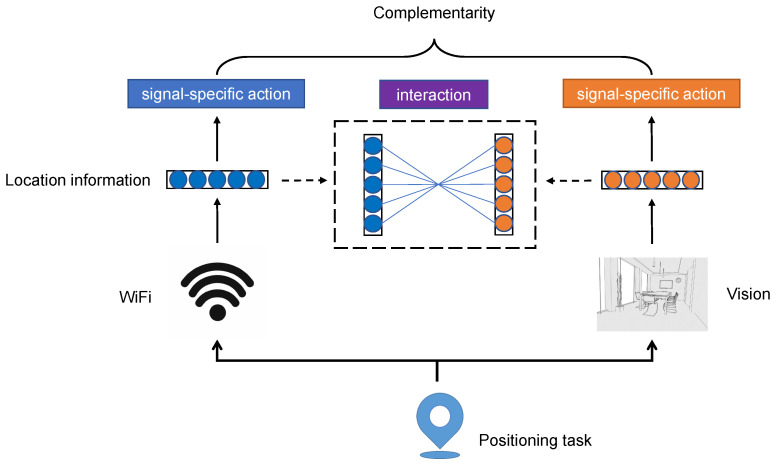
Explanation of the complementarity of WiFi and vision in positioning tasks. We argue that the complementarity of WiFi and vision in positioning tasks are fully reflected in the signal-specific actions and interaction of their location information.

Based on the fact that the signals collected from different locations show different distributions, unique location information can be mined from WiFi or visual signals. Fingerprint localization can be implemented using only one of these two signals, which means that the location information of each signal has an independent positioning action, also called *signal-specific action*; In the field of multimodal learning, modeling the interaction between different modal data has been shown to bring significant performance improvements and has achieved successes in many tasks, such as visual question answering [3] and emotional analysis [4]. We infer that the *interaction* of location information in different signals can also work in the fingerprint localization task. The complementarity of WiFi and visual signals in localization tasks is composed of their signal-specific actions and interaction, as shown in Figure 1. Therefore, to make full use of complementarity to achieve better positioning performance in WiFi and visual fusion localization, a challenge must be faced to effectively model both signal-specific actions and the interaction of location information in the two signals while avoiding complex processes and operations.

Researchers have made some attempts to use the complementarity of WiFi and visual signals for fusion location and made some progress. Some researchers divide the overall fusion positioning process into two stages: rough positioning and fine positioning. Firstly, a rough region is determined by using the location information of one of the signals, and then fine positioning is performed by the other signal on this basis [5,6,7,8]. A similar idea is to first use WiFi and visual signals to locate separately, and then integrate the two results at the decision level according to a certain strategy [9,10]. This kind of method of establishing a separate location model for each signal does not need to consider the heterogeneity of the data, but their process is complex and can not effectively model the interaction between the two signals; Other works attempt to homogenize and fuse the two heterogeneous signals at the data input level [11,12] or feature level [13,14,15]. Inspired by the RGB-D image, Jiao et al. [12] visualize the WiFi data into characteristic images and combine them with the RGB visual images to construct fusion images, called RGB-W images. Then the image feature extractor is used to extract features for fingerprint localization. Refs. [13,14] use different hand-defined or pre-trained feature extractors to extract dimensional unified features of WiFi and visual signal, and then simply splice or bit-by-bit addition of the two features to get fusion fingerprints for location estimation. However, this connection ignores the specific statistical properties of each signal and cannot model the signal-specific actions of location information of different signals. To sum up, the previous works do not account for both signal-specific actions and the interaction of the two signals, so the complementarity was not fully utilized.

**Figure 2:**
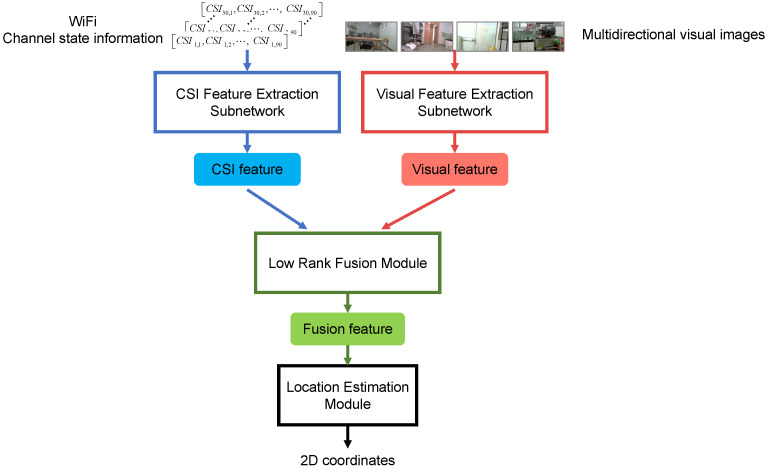
The framework of LRF-WiVi. After offline training, the model can predict the position of the input data in the online stage.

To make full use of the complementarity of heterogeneous signals in indoor localization, this paper proposes an end-to-end WiFi and visual feature level fusion localization model LRF-WiVi, which uses both specific actions and interaction of the two signals. LRFWiVi takes the collected WiFi channel state information (CSI) and multi-directional visual images as input and outputs the position coordinates of the undetermined point. The overall model consists of three modules, as shown in Figure 2. Firstly, in the feature extraction module, two different feature extraction subnetworks are designed to extract and isomorphize the features of two heterogeneous data; Then, in the fusion module, the fusion method based on low-rank factors is used to efficiently fuse the CSI feature and visual feature with low computational complexity, so that the fusion feature can capture both specific actions of the two features and the interaction between them; Finally, the position estimation module estimates the position coordinates of the point to be determined according to the fusion features. The whole model is end-to-end learning, which avoids additional independent training for each module under inconsistent goal constraints.

The rest of this paper is organized as follows: The Section 2 discusses the related work of WiFi channel state information indoor localization, visual indoor localization, and tensor representation fusion. The Section 3 provides details about the proposed model LRF-WiVi, including the feature extraction module, fusion module based on a low-rank factor, and position estimation module. The Section 4 describes the experimental setup and presents the result analysis. The Section 5 summarizes the research and prospects the future work.

## 2. Related Work

**Indoor Localization Based on CSI Characteristic Image.** WLAN devices commonly deployed indoors provide rich signal resources for WiFi-based localization methods, including RSS (Received Signal Strength) and CSI. Compared with RSS, CSI provides the amplitude and phase information of different frequency subcarriers, which can better reflect the characteristics of signal propagation in multipath environments [16,17]. Recent studies have shown that it is feasible to transform the indoor localization problem into the image classification problem by transforming CSI measurement data into characteristic images [18,19,20,21,22]. Chen et al. proposed that ConFi [18] arranges the CSI amplitude values of different subcarriers at different times in the three antenna links into three matrices separately as the three channels of the RGB image to form the CSI characteristic image. Then the convolution neural network is used for classification to realize the localization. Wang et al. proposed that CiFi [21] uses the phase difference of subcarriers between different antennas to estimate AOA (Angle of arrival) and converts it into a single-channel grayscale image to train the classification model based on a depth convolution neural network. The above two methods only use one of CSI amplitude and phase. Li et al. [22] proposed a CSI characteristic image construction method using both amplitude and phase, in which the three channels of RGB images are composed of the subcarrier amplitude of the first antenna, the subcarrier phase difference between the first and second antennas, and the subcarrier phase difference between the second and third antennas. However, the physical meaning of this information combination between different channels is not clear, and only part of the amplitude and phase information of CSI is used. To sum up, the existing localization methods based on CSI characteristic image mostly aim at constructing a characteristic image that can be visualized, which fails to make full use of the rich amplitude and phase information in CSI.

**Indoor Localization Based on Visual Image Matching.** Compared with the visual simultaneous localization and mapping (SLAM) method, the localization method based on image matching has low computational cost and equipment costs, which has aroused the interest of many researchers [23,24,25,26,27]. Such methods need to build an offline database containing the features extracted from the reference images and their corresponding position coordinates. The features of the query image in the online phase are obtained by the same feature extraction method. Then the location matching is realized by comparing the similarity between the feature of the query image and the features in the offline database. The performance of the method based on image matching mainly depends on the discrimination of the extracted image features. Many methods have been applied to scene image feature extraction, including Scale-Invariant Feature Transform (SIFT), Histogram of Oriented Gradient (HOG) [23], Bag-of-Visual-Words (BoVW) [24] and Vector of Locally Aggregated Descriptors (VLAD) [25] based on manually defined image descriptors and Convolutional Neural Network (CNN) [26,27] based on neural networks. In our previous work [14], Shared Convolutional-Neural-Network based Add Fusion Network (SC-AFN) was proposed to extract the key vector feature from multi-directional images and could effectively improve the performance of image matching-based localization.

**Fusion Methods Based on Tensor Representation.** Tensor representation realizes fusion by calculating the outer product of different feature vectors, which has been proven to be more expressive than simple splicing or summation in capturing the interaction between different factors [3,28,29]. The bilinear pooling model proposed by Tenenbaum and Freeman [28] captures the complex interaction between “content” and “style” through the outer product. Lin et al. [29] applied this method to fine-grained visual recognition tasks and proved its effectiveness. To model both intra-modality and inter-modality dynamics, Tensor Fusion Network proposed by Zadeh et al. [4] adds an additional constant dimension with value 1 to the unimodal representations before outer product operation, so that the tensor obtained by the outer product can explicitly aggregate unimodal and multimodal interaction. However, one problem that comes with the strong expressive power of tensor representation is the high number of parameters. The high dimension tensor constructed by the outer product leads to a huge number of parameters to be learned in the weight tensor, bringing a massive computational burden. Some studies use low-rank approximation to make tensor training more efficient and easier to handle, which has been successfully applied to face recognition [30] and multimodal emotion analysis [31] tasks. Therefore, the low-rank approximation of tensor fusion is expected to model unimodal action and multimodal interaction more efficiently with lower parameters.

To fully utilize the rich amplitude and phase information in CSI, this paper proposes a novel CSI characteristic map construction method in the CSI feature extraction subnetwork, which does not limit the number of channels to a single channel of grayscale images or three channels of RGB images for visualization purposes. We take the amplitudes or calibrated phases of N subcarriers in the M consecutive data packets collected from each antenna link as a channel of the CSI characteristic map. This means that for T antenna links, our CSI characteristic map will have 2T channels, in which the first T channels are amplitude characteristic submaps, and the latter T channels are phase characteristic submaps. To effectively mine the amplitude and phase correlation between adjacent subcarriers and different antennas, a double-branch CNN structure is further designed to extract CSI features. Moreover, a visual feature extraction subnetwork based on SC-AFN [14] is designed to extract the unified representation of four azimuth images.

In the feature fusion module, to exploit the specific actions and interaction of two heterogeneous signals in the WiFi and vision fusion fingerprint localization task in a tractable way, this paper proposes a fusion method based on low-rank factors to efficiently aggregate CSI features and visual image features containing specific location information. The obtained fusion features are used for the final location estimation.

## 3. Proposed Method

Our proposed LRF-WiVi consists of three major components: feature extraction module, low-rank fusion module, and position estimation module. The following three sections describe the three modules in detail.

### 3.1. Feature Extraction Module

**CSI feature extraction subnetwork:**

**Figure 3:**
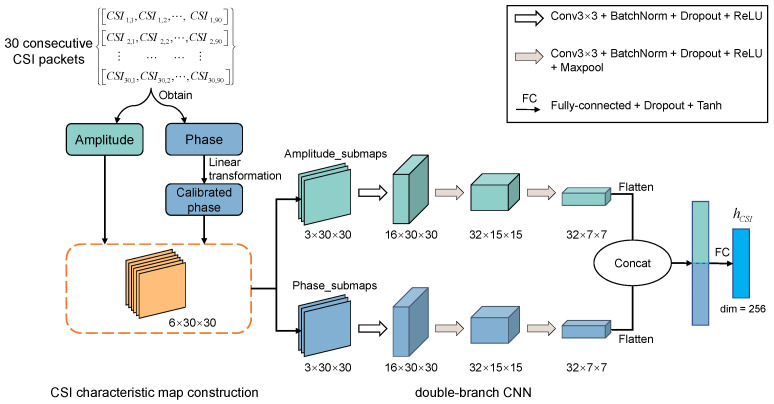
CSI feature extraction subnetwork, including CSI characteristic map construction and a double-branch CNN structure. The network outputs the feature vector containing CSI-specific location information.

The CSI feature extraction subnetwork proposed in this paper includes the construction of a CSI time-frequency characteristic map and a double-branch CNN structure, as shown in Figure 3.

Our proposed CSI characteristic map construction method utilizes the CSI amplitude and phase information of all antenna links, which makes full use of the frequency diversity and spatial diversity of CSI. For M CSI packets collected continuously, each antenna link contains N subcarriers, and the CSI value of each subcarrier is a complex value:

(1) $$csi_{m,n}=\left|csi_{m,n}\right|\exp\left(j∠csi_{m,n}\right)\left(1\leq m\leq M,1\leq n\leq N\right),$$

where $\left|csi_{m,n}\right|$ and $∠csi_{m,n}$ are the amplitude and phase values corresponding to the *n*th subcarrier in the *m*th data packet respectively. For the original phase that cannot be used directly, we use the linear transformation method [32] to process it to obtain the calibrated phase $∠{\hat{csi}}_{m,n}$. Therefore, each antenna link can obtain an amplitude matrix and a phase matrix:

(2) $$AMP=\left[\begin{array}{ccc} \left|csi_{1,1}\right| & \cdots & \left|csi_{1,N}\right|\\ \vdots & \ddots & \vdots \\ \left|csi_{M,1}\right| & \cdots & \left|csi_{M,N}\right| \end{array}\right],PHA=\left[\begin{array}{ccc} ∠{\hat{csi}}_{1,1} & \cdots & ∠{\hat{csi}}_{1,N}\\ \vdots & \ddots & \vdots \\ ∠{\hat{csi}}_{M,1} & \cdots & ∠{\hat{csi}}_{M,N} \end{array}\right],$$

For *T* antenna links, *T* amplitude matrices and *T* phase matrices can be obtained. After min-max normalization, each matrix is used as a channel of the CSI characteristic map. This paper uses the antenna setting of one transmitter and three receivers, so the number of antenna links is T=1×3. For M=N=30, we get the time-frequency characteristic map of CSI with the size of 6×30×30, in which the first three channels are amplitude submaps and the last three channels are phase submaps.

We design a double-branch CNN to extract the feature vector containing location information from the CSI characteristic map. The amplitude submaps and phase submaps are processed by two branch networks with the same structure respectively. Each branch network includes convolution, BatchNorm, Dropout, Maxpool, and ReLu activation layers. The convolution operation can mine the correlation of amplitude or phase of adjacent subcarriers and combine the information between different antennas. A small probability Dropout (the probability in this paper is set to 0.1) is used between different convolution layers to increase the robustness of the model to noise input. The amplitude and phase intermediate features obtained by the two branches are respectively expressed as $feature_{amp}$ and $feature_{pha}$:

(3) $$\begin{array}{cc} \; & feature_{amp}=Amplitude\_branch\left(\left[\;\mathrm{Amplitude}\_\mathrm{submaps}\right]\right),\\ \; & feature_{pha}=Phase\_branch\left(\left[\;\mathrm{Phase}\_\mathrm{submaps}\right]\right), \end{array}$$

where Amplitude_submaps and Phase_submaps are three-channel amplitude submaps and three-channel phase submaps corresponding to three antenna links, respectively. Amplitude_branch and Phase_branch are two branch networks with the same structure but not shared parameters. The two intermediate features are flattened and spliced, and then integrated through a full connection layer:

(4) $$h_{CSI}=\mathrm{FC}\_\mathrm{layer}\left(\left[\mathrm{Flatten}\left({feature}_{amp}\right),\mathrm{Flatten}\left({feature}_{pha}\right)\right]\right).$$

Let $h_{CSI}$ denote the CSI feature vector obtained by the CSI feature extraction subnetwork, whose size is 256.

**Visual feature extraction subnetwork:**

**Figure 4:**
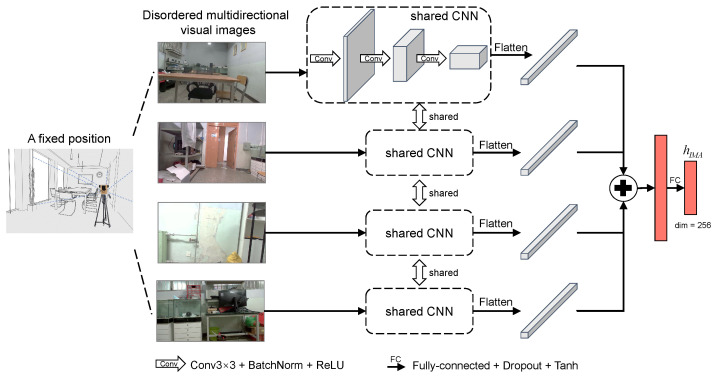
Visual feature extraction subnetwork based on SC-AFN. The network outputs feature vectors that contain specific location information of the visual images.

Compared with unidirectional images, multi-directional images can capture more abundant visual information and alleviate the loss of location information caused by indoor objects or walls obscuring the camera to some extent. Our previous work [14] proposed SC-AFN to extract the unified representation of multi-directional visual images. In this paper, a visual feature extraction subnetwork is designed based on SC-AFN, as shown in Figure 4. The scene images of four directions collected at the same point are fed into the network in disorder. The size of the visual feature vector obtained is consistent with that of CSI feature vectors, which are both 256.

### 3.2. Low-Rank Fusion Module

**Figure 5:**
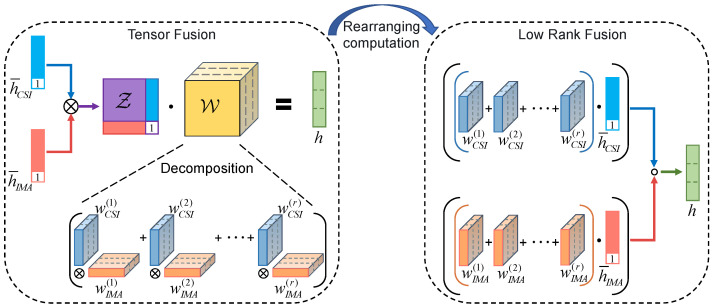
Derivation of low-rank fusion method. Left: The introduction and approximate decomposition of Tensor Fusion. Right: Low-Rank Fusion based on signal-specific low-rank factors.

To capture both the signal-specific actions and interaction of location information in WiFi and visual signals, the low-rank fusion method is introduced to fuse the CSI feature and visual image feature. Figure 5 shows the derivation process from the tensor fusion method to the low-rank fusion method.

**Tensor Fusion Method:**

The tensor fusion method can explicitly model unimodal and multimodal interaction by outer product. The fusion process of the CSI feature vector and visual feature vector based on this method is shown in the dotted frame on the left side of Figure 5.

${\bar{h}}_{CSI}$ and ${\bar{h}}_{IMA}$ represent the CSI feature vector and visual feature vector with an additional constant dimension of value 1, respectively:

(5) $${\bar{h}}_{CSI}=\left[\begin{array}{c} h_{CSI}\\ 1 \end{array}\right],{\bar{h}}_{IMA}=\left[\begin{array}{c} h_{IMA}\\ 1 \end{array}\right].$$

The tensor obtained from the outer product of ${\bar{h}}_{CSI}$ and ${\bar{h}}_{IMA}$ can be expressed as:

(6) $$Z={\bar{h}}_{CSI}⊗{\bar{h}}_{IMA}=\left[\begin{array}{cc} h_{CSI}⊗h_{IMA}\; & h_{CSI}\\ h_{IMA}^{T}\; & 1 \end{array}\right],$$

where ⊗ represents the outer product between vectors, $h_{CSI}$ and $h_{IMA}^{T}$ in the tensor corresponding to the location information in a single signal, and $h_{CSI}⊗h_{IMA}$ forms the interaction between location information of CSI and multi-directional images. Then the outer product tensor is passed through a linear layer to produce a fusion representation:

(7) h=W·Z+b,

where W is the weight tensor of the linear layer, and *b* is the bias. When the size of ${\bar{h}}_{CSI}$ and ${\bar{h}}_{IMA}$ are both 257, $Z\in R^{257\times 257}$, $W\in R^{257\times 257\times d_{h}}$ and $b\in R^{d_{h}}$. $d_{h}$ is the size of fusion representation vector *h*.

The fusion representation obtained by the tensor fusion method can capture both signal-specific actions and the interaction of location information in the two signals. However, the number of parameters of the weight tensor W is relatively large. This not only makes the model training difficult but also has the risk of over-fitting.

**Low Rank Fusion Method:**

To further reduce the parameters and computational complexity of the fusion model while capturing the signal-specific actions and interaction of location information in WiFi and visual signals, we decompose the weight tensor W into a set of signal-specific low-rank factors. The last dimension of the third-order tensor $W\in R^{257\times 257\times d_{h}}$ corresponds to the fusion representation of the output, and the first two dimensions correspond to the feature vectors of the two signals respectively. Approximate decomposition is only performed on the first two dimensions, which can be expressed as:

(8) $$W\approx \sum_{i=1}^{r}w_{CSI}^{\left(i\right)}⊗w_{IMA}^{\left(i\right)},$$

where $w_{CSI}^{\left(i\right)}\in R^{257\times d_{h}}$ and $w_{IMA}^{\left(i\right)}\in R^{257\times d_{h}}$ are low-rank factors specific to CSI and visual images. The outer product in Equation (8) is performed only on dimensions other than the last dimension. ${\left\{w_{CSI}^{\left(i\right)},w_{IMA}^{\left(i\right)}\right\}}_{i=1}^{r}$ are the rank *r* decomposition factors of the weight tensor. For a fixed rank *r*, we parameterize *r* decomposition factors that can reconstruct the low-rank version of the weight tensor.

In addition, using the fact that $Z={\bar{h}}_{CSI}⊗{\bar{h}}_{IMA}$, Equation (7) can be simplified to Equation (9) (the bias *b* is ignored here):

(9) $$\begin{array}{cc} h & =W\cdot Z\\ \; & =\sum_{i=1}^{r}\left(w_{CSI}^{\left(i\right)}⊗w_{IMA}^{\left(i\right)}\right)\cdot \left({\bar{h}}_{CSI}⊗{\bar{h}}_{IMA}\right)\\ \; & =\left(\sum_{i=1}^{r}w_{CSI}^{\left(i\right)}\cdot {\bar{h}}_{CSI}\right)\circ \left(\sum_{i=1}^{r}w_{IMA}^{\left(i\right)}\cdot {\bar{h}}_{IMA}\right), \end{array}$$

where ∘ represents the element-wise product between tensors. Because Equation (9) is differentiable, the parameters can be learned by backpropagation.

The low-rank fusion method based on Equation (9) is shown in the dotted frame on the right side of Figure 5. This method avoids the creation of tensors by the outer product and the subsequent linear projection of high weights. Instead, the fusion feature *h* can be calculated directly from ${\bar{h}}_{CSI}$,${\bar{h}}_{IMA}$, and signal-specific low-rank factors. The low-rank fusion method reduces the computation complexity of fusion from $O(257\times 257\times d_{h})$ to $O(r\times \left(257+257\right)\times d_{h})$. Our experiments show that superior performance can be achieved with a very small value (i.e., 2≪257) (see Section 4.3.3). Therefore, the low-rank fusion method can aggregate the specific location information contained in CSI features and visual features and the interaction between them in a more efficient way.

### 3.3. Position Estimation

In this paper, indoor localization is defined as a multi-classification problem of reference points (RPs). Since we focus on the impact of fusion methods on positioning performance, a simple softmax classifier is used to classify the input fusion features in the position estimation module.

We set the size of the fusion feature to be equal to the number of RPs (i.e., $d_{h}=num\_RPs$). The probability that h belongs to the *i*th RP in the output of the softmax classifier can be expressed as:

(10) $$\mathrm{softmax}{\left(h\right)}_{i}=\frac{e^{h_{i}}}{\sum_{j}^{num\_RPs}e^{h_{j}}},$$

where $h_{i}$ represents the *i*th eigenvalue in the fusion feature vector.

To train our model end-to-end in the offline phase, cross-entropy is adopted as the overall loss function, which is formulated as:

(11) $$L=-\sum_{i}^{}y_{i}log\left({\hat{y}}_{i}\right),$$

where $\hat{y}$ is the predicted probability distribution and *y* is the real probability distribution. The Adam optimization algorithm is adopted to iteratively update the network parameters to minimize the loss function to complete the training of the model.

In the online positioning phase, the CSI and visual images collected by the equipment are input into the trained model. The model will output the probability values of the positioning device at each RP. We select the three RPs with the largest probability, and obtain the final position estimation by the weighted centroid method:

(12) $$L=\sum_{i=1}^{3}\frac{p_{i}L_{i}}{\sum_{j=1}^{3}p_{j}},$$

where *L* is the final predicted coordinate, $p_{1}$, $p_{2}$, and $p_{3}$ are the three maximum probability values of the model output, and $L_{1}$, $L_{2}$, and $L_{3}$ are the coordinates of RPs corresponding to the three probability values respectively.

## 4. Experiments

To evaluate the performance of our proposed LRF-WiVi, we design the following four experiments:

**(1) Evaluation of CSI feature extraction subnetwork:** We compare the proposed CSI feature extraction subnetwork with other feature extraction methods based on CSI images.

**(2) Comparison between single-source localization models and multi-source fusion localization model:** We connect the CSI feature extraction subnetwork with the position estimation module through a full connection layer to obtain a CSI-based single-source localization model. The same method is used to get the vision-based single-source location model. The two single-source localization models are compared with our fusion localization model.

**(3) Impact of rank setting:** We compare and analyze the performance of LRF-WiVi under different rank settings.

**(4) Comparison of different fusion methods:** We compare the proposed model with the localization models based on other fusion methods, including stage-based [5], decisionlevel threshold-based [9], feature-level splicing [13], and tensor fusion [4]. The purpose of this experiment is to explore the impact of different fusion methods on positioning performance. Therefore, to ensure the fairness and validity of the comparison, the same means of feature extraction and prediction probability output were used in different localization models except for fusion methods.

### 4.1. Experimental Environment and Datasets

**Figure 6:**
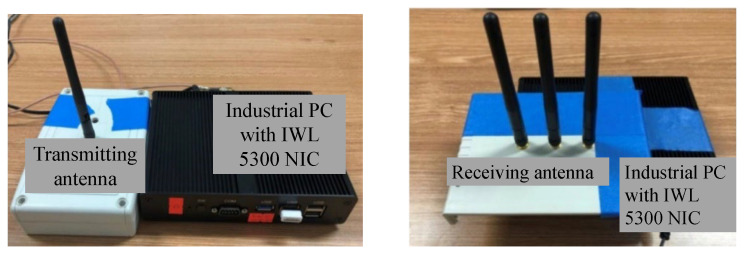
CSI data collection equipment. The mobile terminal has a transmitting antenna (**left**) and the fixed terminal has three receiving antennas (**right**).

**Figure 7:**
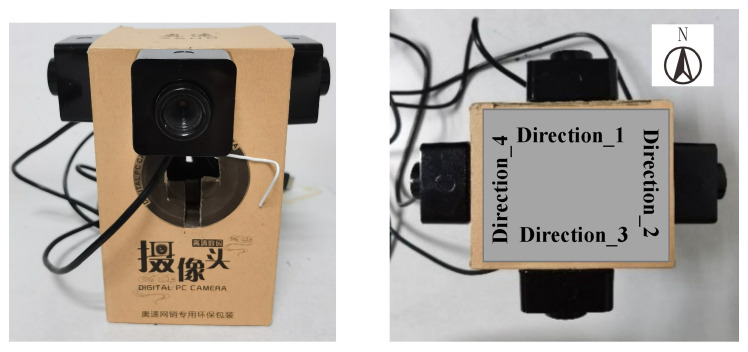
Multi-directional images collection equipment.((**Left**): front view, (**Right**): top view).

We collected data in a small laboratory with a complex environment and a relatively empty elevator hall. It is argued that experiments in these two scenarios can fully evaluate the performance of the localization model in different environments. Data collection includes CSI collection of the WiFi signal and multi-directional image collection of the visual signal. CSI data collection equipment consists of a transmitter and a receiver, both of which are industrial computers with built-in Intel 5300 wireless network cards, as shown in Figure 6. The transmitter with one antenna is used as the mobile terminal, continuously sending data packets under the 20 MHz bandwidth. The receiver with three antennas is placed in a fixed position indoors and can receive and store corresponding data packets. The visual images data collection equipment is built by four monocular cameras, as shown in Figure 7. It can simultaneously collect four scene images in different directions through the control of the mobile terminal.

The selection of RPs is a necessary part of fingerprint location. Some previous studies have analyzed the influence of the number or interval of reference points on positioning accuracy. Cheng et al. [33] concluded through experiments that the more RPs (the smaller the interval), the smaller the fingerprint positioning error. The research of Bahl et al. [34] showed that when the number of RPs exceeds a certain threshold, the positioning error almost no longer decreases. However, Wang et al. [35] argued that the positioning error would increase when the number of RPs exceeds the maximum set of reference points due to the existence of noise. The experiment of Zhang et al. [36] also showed a similar pattern to [35]. Too small intervals would not help to improve positioning accuracy, but would only increase the labor and time of building the fingerprint database. On the other hand, too large intervals would not be beneficial to improve the positioning accuracy, too.

In this work, we select the locations of the RPs evenly at appropriate intervals considering both positioning accuracy and data collection labor. The distance between the adjacent RPs in the laboratory is 0.8 m, while that in the hall is 0.93 m. Our settings ensure good positioning accuracy and make the workload of offline data collection acceptable.

**Figure 8:**
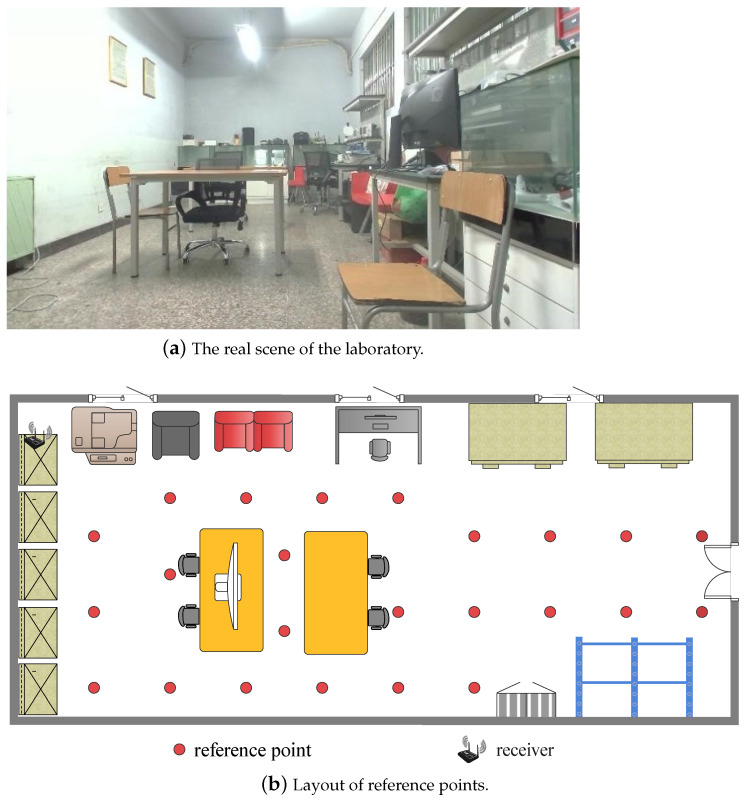
The diagram of the laboratory environment.

**The laboratory environment** is located in Room 523 on the fifth floor of the main building of Beijing University of Posts and Telecommunications. Obstacles in the room include desks, chairs, showcases, computers, and other experimental equipment, as shown in Figure 8a. The area of the laboratory is about 4.0 m × 8.0 m. 24 reference points are set, and their distribution is shown in Figure 8b. The WiFi receiver is fixed above the 1.7 m high showcase and is close to the wall. The height of the transmitter on the mobile end is 0.4 m, which is lower than the height of tables, chairs, showcases, and other obstacles. Therefore, the WiFi signal at most of the reference points in this scene is propagated in non-line-of-sight. We collected CSI and multi-directional images of each reference point in two different time periods. There were some changes in the distribution of indoor obstacles between the two periods. Data samples of quiet and random walking were collected in each time period, and each sample was composed of 30 consecutive CSI data packets and a set of multi-directional images. The data collected in the first time period is used as the offline training set, and the number of training samples at each reference point is 40. The data collected in another period is used as an online test set, and the number of test samples at each reference point is 40.

**Figure 9:**
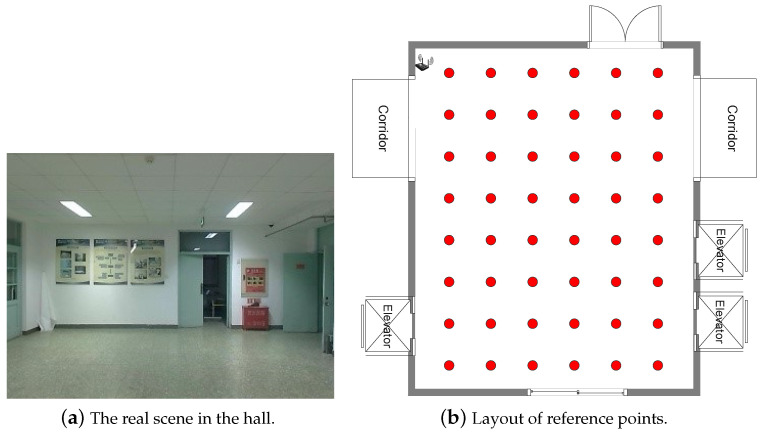
The diagram of the hall environment.

**The hall environment** is located on the fifth floor of the main building of Beijing University of Posts and Telecommunications. There are no obvious obstacles except for the movement of people in the scene, as shown in Figure 9a. The area of the hall is 6.2 m × 8.0 m. 48 reference points are set and the distance between the adjacent reference points is 0.93 m. Their distribution is shown in Figure 9b. The WiFi receiver is fixed on the wall 2.4 m above the ground, and the height of the transmitter on the mobile end is also 0.4 m. Therefore, the WiFi signal is mainly propagated by line-of-sight in the hall. We collected data in two different time periods in the same way as in the laboratory environment. The data collected in the first time period is used as the offline training set, and the number of training samples at each reference point is 40. The data collected in another time period is used as the online test set, and the number of test samples at each reference point is 4.

### 4.2. Evaluation Metrics

To comprehensively analyze the positioning performance of the model, we consider the following evaluation metrics in the evaluation process: positioning mean error, standard deviation, and the number of fusion module parameters.

For test sample *i*, the positioning error represents the distance between the predicted coordinate and the real coordinate, which is calculated as follows:

(13) $${error}_{i}=\sqrt{{\left(x_{i}-{\hat{x}}_{i}\right)}^{2}+{\left(y_{i}-{\hat{y}}_{i}\right)}^{2}},$$

where $\left(x_{i},y_{i}\right)$ is the true coordinate of test sample *i*, and $\left({\hat{x}}_{i},{\hat{y}}_{i}\right)$ is the coordinate predicted by the localization model.

For all the test samples, the positioning mean error and standard deviation are respectively calculated by

(14) $$Mean=\frac{1}{N}\sum_{i=1}^{N}{error}_{i},$$

(15) $$Std=\sqrt{\frac{1}{N}\sum_{i=1}^{N}{\left({error}_{i}-Mean\right)}^{2}},$$

here *N* is the total number of test samples.

The positioning mean error and standard deviation reflect the positioning accuracy and stability of the model respectively. The number of parameters reflects the computational complexity of the model. For all the above metrics, lower values denote better performance. In addition, the error cumulative distribution function (CDF) curve is also used to show the distribution of the positioning errors of test samples.

### 4.3. Experimental Results and Analysis

#### 4.3.1. Evaluation of CSI Feature Extraction Subnetwork

**Figure 10:**
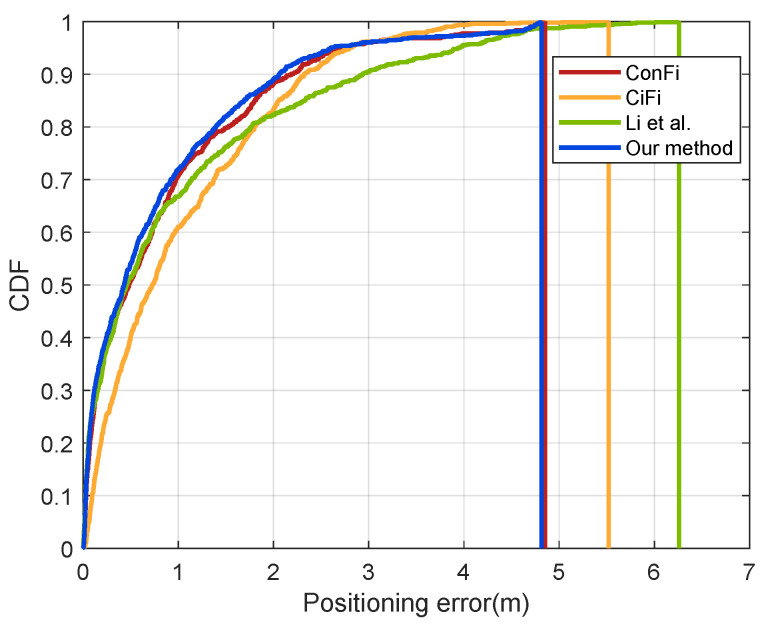
The CDF of positioning errors for different CSI extraction methods [18,21,22].

To verify the performance of our CSI feature extraction subnetwork, we compared it with three other feature extraction methods based on CSI images, including ConFi [18], CiFi [21] and the method proposed by Li et al. [22]. Different methods were used to extract CSI features for fingerprint localization in the laboratory. The results are shown in Table 1 and Figure 10.

The comparison of positioning results shows that the overall performance of our CSI feature extraction method is better than the other three methods. Compared with ConFi only using amplitude information, CiFi only uses phase information, and the method of Li uses partial amplitude and phase information, our method combines all amplitude and phase information to make full use of the frequency diversity and spatial diversity characteristics of CSI. The double-branch CNN structure can extract and integrate the location information contained in the amplitude and phase of all antenna links, allowing better modeling of the specific positioning action of CSI.

#### 4.3.2. Comparison with Single-Source Localization Model

**Figure 11:**
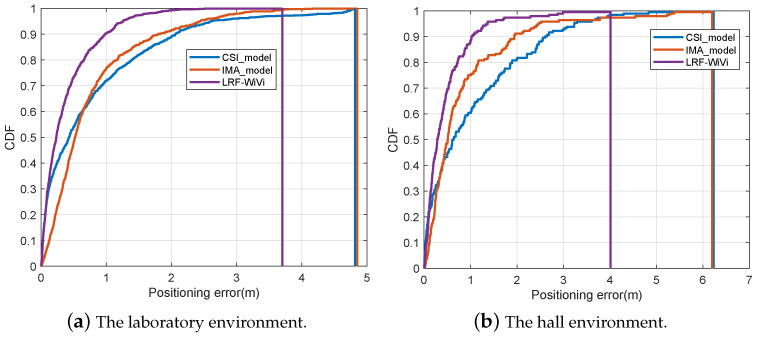
Comparison of positioning errors distribution with single-source model.

In this experiment, we compare our fusion localization model with the single-source localization models constructed by using a single signal feature extraction subnetwork. The experimental results on the datasets of the two environments are shown in Table 2 and Figure 11.

Table 2 shows that the proposed LRF-WiVi is significantly better than the single-source localization models in terms of positioning mean error and standard deviation. In the case of the laboratory environment, the positioning mean error of LRF-WiVi is 0.38 m, 51.90% lower than 0.79 m of the CSI localization model, and 50.65% lower than 0.77 m of the visual localization model. The positioning standard deviation of LRF is 0.43 m, 56.12% lower than 0.98 m of the CSI localization model and 42.67% lower than 0.75 m of the visual localization model; The CDFs shown in Figure 11 shows that the CSI model is better than the image model in the short distance error interval (the left side of the intersection of the two curves), and worse than the image model in the medium and long-distance error interval. The two signals have their own advantages and disadvantages. LRF-WiVi has better positioning performance than the two single-source models in all error ranges, and greatly reduces the maximum positioning error. The above experimental results show that LRF-WiVi can effectively make use of the complementarity of location information in the two signals, to achieve higher positioning accuracy and stability.

#### 4.3.3. Impact of Rank Setting

**Figure 12:**
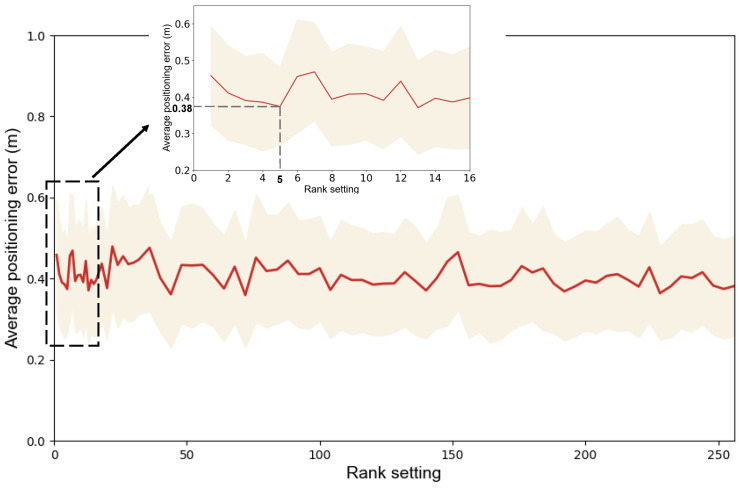
The impact of different rank settings on positioning performance. We select different verification intervals in different ranges of rank, with intervals of 1 in [1, 16], 2 in [16, 32], and 4 in [32, 256]. In the figure, the result of setting rank at [1, 16] is enlarged.

To evaluate the impact of different rank settings on the LRF-WiVi positioning performance, we measure the change in performance on the laboratory dataset while varying the number of ranks. The results are shown in Figure 12, where the points on the red curve represent the positioning mean error of the model under the current rank setting, while the light-shaded part reflects the change of the positioning stability of the model to some extent (better visualization by scaling the standard deviation).

It could be observed that with the increase of rank, the positioning mean error does not show a smooth downward trend, but keeps fluctuating in a small range. And a very small rank is enough to obtain excellent positioning performance. For the comprehensive consideration of positioning accuracy, stability, and model complexity, we set the rank of LRF-WiVi to be 5 in other experiments.

#### 4.3.4. Comparison with Other Fusion Methods

**Figure 13:**
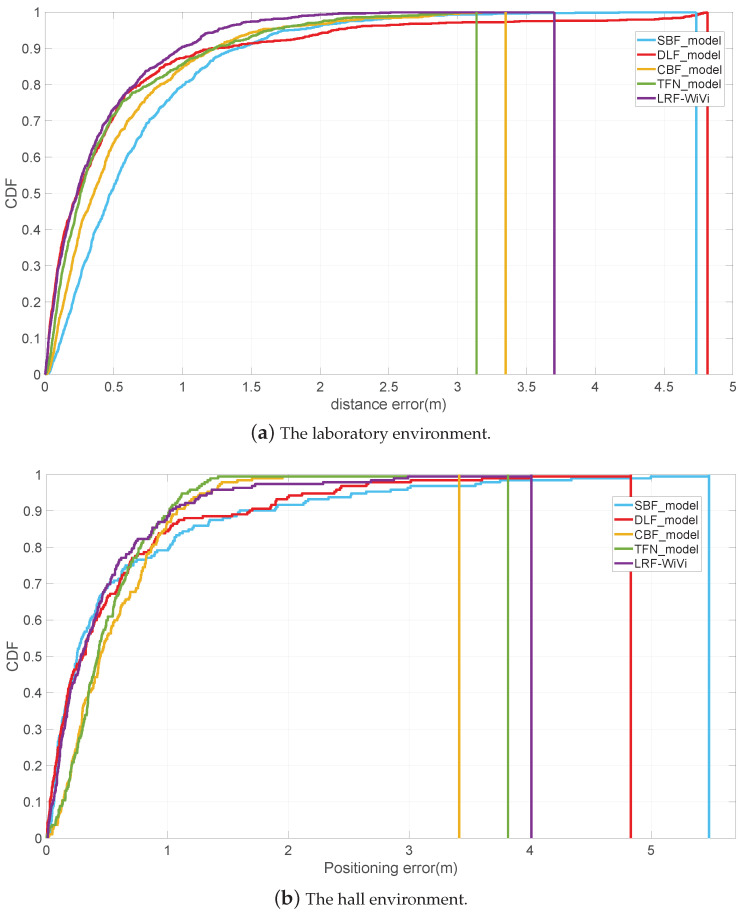
Comparison of positioning errors distribution of different fusion methods.

To evaluate the effectiveness of the low-rank fusion method, we compare LRF-WiVi with localization models based on other fusion methods, including stage-based (SBF) [5], decision-level threshold-based (DLF) [9], feature-level splicing (CBF) [13] and tensor fusion network (TFN) [4]. The experimental results on the datasets of the two environments are shown in Table 3 and Figure 13.

Figure 13 shows the CDFs of positioning errors of different models on the online test set. The results of the laboratory environment are shown in Figure 13a. The third quartile of LRF-WiVi positioning errors is 0.54 m (that is, the positioning errors of 75% of the test samples are less than 0.54 m), which is 38.64%, 1.82%, 22.86% and 3.57% lower than 0.88 m of SBF, 0.55 m of DLF, 0.70 m of CBF and 0.56 m of TFN respectively; The results of the hall environment are shown in Figure 13b. The third quartile of LRF positioning errors is 0.59 m, which is 18.06%, 14.49%, 27.16% and 15.71% lower than 0.72 m of SBF, 0.69 m of DLF, 0.81 m of CBF and 0.70 m of TFN respectively.

Table 3 shows the comparison of different models in terms of positioning mean error, standard deviation, and the number of fusion module parameters. In the laboratory dataset, LRF-WiVi achieves the best positioning accuracy and stability, and its positioning mean error and standard deviation are lower than those of other fusion methods. The performance of TFN is only inferior to LRF, and it is also superior to the other three fusion methods. The results show that modeling both signal-specific actions and the interaction of the two signals can improve the positioning performance of the model. While LRF-WiVi can further improve the accuracy and stability on the basis of reducing the number of parameters compared with TFN, indicating that the modeling of LRF-WiVi is more efficient; In the hall dataset, the positioning mean error of LRF is 0.47 m, and the standard deviation is 0.55 m. Compared with other methods, LRF-WiVi has the best positioning accuracy, but its stability is slightly worse than TFN and CBF. In terms of computational complexity, the method with the least number of parameters is CBF, which is 0.02 M, because it only performs simple splicing. The parameter quantity of the LRF-WiVi fusion module is 0.06 M, slightly higher than that of CBF. TFN with the best positioning stability has 17.12 M parameters, which is much larger than that of LRF and CBF. Therefore, considering the results of the three metrics, we can still conclude that LRF-WiVi has the best performance.

To sum up, the performance of LRF-WiVi in both laboratory and hall environments is better than the localization models based on other fusion methods. It proves that LRF-WiVi can make full use of the complementarity of the two signals. In addition, we note that the results in Figure 13b show that the performance of TFN is better than that of LRF-WiVi when the positioning error distance is greater than 1m. Our analysis of this is as follows. In the hall, the distribution difference between the test data and the training data on some RPs is very small because there is no change of obstacles between the two data collections except for the random walk of people. For such RPs, the TFN with a large number of parameters fits better (even over-fits) and can suppress long-range mismatching better than LRF-WiVi. In the laboratory, due to the change in the location of indoor obstacles between the two data collections, there are some distribution differences between the test data and training data of each RP. In this case, LRF-WiVi outperforms TFN for almost all tested samples, as shown in Figure 13a. We believe that the difference between the results in the two experimental scenarios proves to some extent that LRF-WiVi has a stronger generalization ability than TFN.

## 5. Conclusions

To make full use of the complementarity of location information in WiFi and visual signals, this paper proposes a localization model based on low-rank fusion, which can model the signal-specific actions and interaction of CSI and multi-directional images end-to-end, avoiding complex training processes. The whole model consists of a feature extraction module, a low-rank fusion module, and a position estimation module. The feature extraction module takes the CSI data packets collected continuously and a group of unordered multi-directional images as input. Two feature extraction subnetworks are designed to extract feature vectors containing CSI and visual image location information respectively. The low-rank fusion module fuses the feature vectors of the two signals efficiently through the signal-specific low-rank factors of the signal. Finally, the position estimation module outputs the position coordinates according to the fusion features. The experimental results show that the performance of our LRF-WiVi is much better than that of the single-source localization models. Compared with the localization models based on other fusion methods, our model also has quite competitive performance. The positioning mean errors in the laboratory and hall scenes are 0.38 m and 0.47 m respectively.

However, the time interval between different data collections in this work was short, and there was no significant change in indoor obstructions. Therefore, despite the presence of random movement of people, the distribution difference between offline databases and online data is limited. Although this accords with the basic hypothesis of traditional fingerprint localization, it still faces the severe test brought by the drastic change of indoor environment in practical application. In future work, we will study the domain-invariant fingerprint feature extraction method under multiple spatio-temporal conditions, to achieve long-term robust localization in complex and highly dynamic indoor environments.

## Figures and Tables

**Table 1:** Comparison of different CSI feature extraction methods. Although the positioning standard deviation of our method is slightly higher than that of CiFi, the positioning mean error of our method is much lower than that of CiFi.

Feature Extraction Method	CSI Information	Mean(m)	Std(m)
ConFi	Amplitude	0.84	1.00
CiFi	Phase	1.02	0.96
Li et al.	Partial amplitude and phase	1.00	1.26
Our CSI subNetwork	All amplitude and phase	0.79	0.98

**Table 2:** Comparison of positioning mean error and standard deviation with single-source models.

Localization Model	Laboratory		Hall	
	Mean(m)	Std(m)	Mean(m)	Std(m)
CSI_model	0.79	0.98	1.05	1.13
IMA_model	0.77	0.75	0.84	1.02
LRF-WiVi	0.38	0.43	0.47	0.55

**Table 3:** Comparison of different fusion methods. For the fusion method using a neural network, the number of parameters of the corresponding fusion module is also given.

Fusion Method	Laboratory		Hall		Fusion Module Param (M)
	Mean (m)	std (m)	Mean (m)	std (m)	
SBF	0.65	0.59	0.63	0.92	N/A
DLF	0.53	0.85	0.57	0.77	N/A
CBF	0.53	0.55	0.56	0.43	0.02
TFN	0.47	0.54	0.52	0.40	17.12
LRF(ours)	0.38	0.43	0.47	0.55	0.06

## Data Availability

Not applicable.
